# Microlipophagy from Simple to Complex Eukaryotes

**DOI:** 10.3390/cells14020141

**Published:** 2025-01-18

**Authors:** Ravinder Kumar, Colin Arrowood, Micah B. Schott, Taras Y. Nazarko

**Affiliations:** 1 Department of Clinical Pharmacy and Translational Science, College of Pharmacy, University of Tennessee Health Science Center, Memphis, TN 38163, USA; fravinde@uthsc.edu; 2 Department of Biology, Georgia State University, Atlanta, GA 30303, USA; carrowood1@gsu.edu; 3 Department of Biochemistry and Molecular Biology, University of Nebraska Medical Center, Omaha, NE 68198, USA; mschott@unmc.edu

**Keywords:** autophagy, chaperon-mediated autophagy, lipid droplets, lipophagy, macroautophagy, macrolipophagy, microautophagy, microlipophagy, selective autophagy, vacuolar microdomains

## Abstract

Lipophagy is a selective degradation of lipid droplets in lysosomes or vacuoles. Apart from its role in generating energy and free fatty acids for membrane repair, growth, and the formation of new membranes, lipophagy emerges as a key player in other cellular processes and disease pathogenesis. While fungal, plant, and algal cells use microlipophagy, the most prominent form of lipophagy in animal cells is macrolipophagy. However, recent studies showed that animal cells can also use microlipophagy to metabolize their lipid droplets. Therefore, to no surprise, microlipophagy is conserved from simple unicellular to the most complex multicellular eukaryotes, and many eukaryotic cells can operate both forms of lipophagy. Macrolipophagy is the most studied and better understood at the molecular level, while our understanding of microlipophagy is very sparse. This review will discuss microlipophagy from the perspective of its conservation in eukaryotes and its importance in diseases. To better appreciate the conserved nature of microlipophagy, different organisms and types of cells in which microlipophagy has been reported are also shown in a tabular form. We also point toward the gaps in our understanding of microlipophagy, including the signaling behind microlipophagy, especially in the cells of complex multicellular organisms.

## 1. Introduction

Autophagy is a process through which cells turn over their own components to restore or maintain their homeostasis. In autophagy, cell components are degraded in lysosomes (in animal cells) and vacuoles (in the case of protozoa, fungi, plants, and algae). Cellular components that are degraded through autophagy may include surplus, damaged, or nonfunctional organelles, proteins, protein complexes, protein aggregates, and foreign entities [1]. Over the years, dozens of autophagic pathways have been identified based on the cellular component being degraded in lysosomes/vacuoles. For example, the degradation of yeast mitochondria (mitophagy) [2,3], peroxisomes (pexophagy) [4,5], lipid droplets (lipophagy) [6,7], and so on. Highlighting all the autophagic pathways is beyond the scope of the present review and can be found elsewhere [8]. The most recent addition to the list of yeast autophagic pathways is glycophagy in *Saccharomyces cerevisiae* (where glycogen granules are selectively degraded in the vacuole) [9].

The conservation of autophagic pathways and associated genes from unicellular yeast to multicellular organisms (including plants, invertebrates, and vertebrates) suggests that autophagy is at the heart of all eukaryotic cells, and eukaryotic cells might have acquired this process quite early in evolution [10]. Besides regulating cellular homeostasis, autophagy has been associated with diverse cell or organ-specific functions and clinical conditions. For example, research over the years has implicated autophagy in development and differentiation (reviewed in [11]), cell cycle regulation [12], morphogenesis and stem cell maintenance [13], apoptosis [14] and non-apoptotic cell death pathways [15], sexual reproduction [16], and immunity and inflammation [17]. Apart from involvement in normal physiology, autophagy is also associated with several disease conditions, including cancer [18], obesity and diabetes [19], liver [20] and neurodegenerative [21] diseases, and microbial infections, including viral [22] and bacterial [23].

It is important to mention that all the known autophagic pathways fall within three broad categories, namely macroautophagy, microautophagy, and chaperone-mediated autophagy (CMA). In macroautophagy, the cellular component is first separated and enclosed in a double membrane structure referred to as an autophagosome, which later fuses with the lysosome to form the autolysosome where the degradation of autophagic cargo takes place [24]. In the case of microautophagy, autophagic cargo is directly taken up by the lysosome/vacuole (reviewed by [25]). Based on morphological changes in the lysosomal/vacuolar membrane, microautophagy can be divided into three subtypes, namely lysosomal/vacuolar protrusion, lysosomal/vacuolar invagination, and endosomal invagination [26,27]. Finally, in CMA, cellular components, primarily proteins having KFERQ peptide sequence, are first recognized by the cell chaperone, Hsc70, before moving into the lysosome for degradation [28,29]. Apart from these three types of autophagy, there is a specialized kind of autophagy known as crinophagy. In crinophagy, secretory granules fuse with lysosomes, and the content of secretory granules is degraded in lysosomes. This type of autophagy is common in the beta cells of the pancreas [30,31]. A detailed discussion on autophagy is beyond the scope of this review and can be found elsewhere [32,33,34].

Our understanding of macroautophagy pathways is much better than microautophagy pathways, including microlipophagy. Different macroautophagic pathways have been described in detail over the years again and again. This review will focus mainly on microlipophagy and how this process is conserved from unicellular yeast to complex multicellular mammals. We will proceed first by briefly discussing lipophagy in general, followed by a detailed discussion of microlipophagy and how it contributes to cell physiology and pathology. Finally, we will identify the current gaps in knowledge about this fascinating and important microautophagic process.

## 2. Lipophagy

As stated above, lipophagy means lysosomal or vacuolar selective degradation of lipid droplets (lipid bodies, lipid particles, or oil bodies). Before lipophagy was discovered in 2009, lipolysis was the only known means of lipid droplet (LD) degradation [35]. Since then, lipophagy as a pathway of LD degradation has been reported in diverse species, including yeast (*S. cerevisiae* and *K. phaffii*) [6,7,36], mice (*Mus musculus*) [35,37,38], fish (*Danio rerio*) [39,40,41], green algae (*Chlamydomonas reinhardtii*) [42], worms (*Caenorhabditis elegans*) [43], fly (*Drosophila melanogaster*) [44], and plants (*Arabidopsis thaliana*) [45]. Examples of species showed that the lipophagy pathway is conserved across the eukaryotic domain of life, from simple yeast to complex multicellular plants and animals. We avoid highlighting the importance of LDs and lipophagy as these aspects have been discussed separately [46,47,48]. For the same reason, we skip the discussion on LD composition, structure, and distribution [49,50,51].

**Figure 1:**
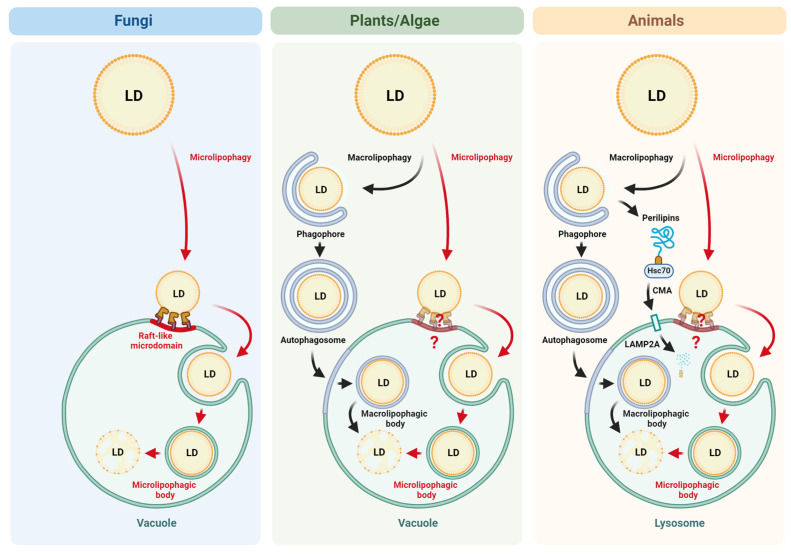
Lipophagy from fungi to higher eukaryotes. Lipophagy in fungi occurs through microlipophagy. Under stress conditions, LDs are transported to the vacuolar membrane. At the vacuole, LDs interact with the raft-like vacuolar microdomains. Then, LDs are internalized by the vacuole, and microlipophagic bodies are formed inside the vacuole before their disintegration by vacuolar hydrolases. Lipophagy in plants and algae occurs through microlipophagy as well as macrolipophagy. Macrolipophagy involves a cup-like phagophore, which is an intermediate of the double-membrane autophagosome that encircles and transports LD to the vacuole. Fusion of the autophagosome with the vacuole generates the macrolipophagic body inside the vacuole, which is decomposed by vacuolar hydrolases. The extent to which plants and algae form vacuolar microdomains for microlipophagy is not yet known. Lipophagy in animals occurs through microlipophagy and macrolipophagy, which is assisted by the CMA pathway. Several LD-associated perilipins must be degraded by Hsc70- and LAMP2A-mediated CMA before the LD can be degraded by macrolipophagy. The extent to which animals form microdomains for microlipophagy is currently unknown. Created in BioRender. Arrowood, C. (2025) https://BioRender.com/m15y166 (accessed on 30 November 2024).

So far, two forms of lipophagy have been reported, namely macro- and microlipophagy [46]. In macrolipophagy (Figure 1, middle and right panels), LDs are (i) surrounded by an isolation membrane (phagophore) followed by (ii) complete sequestration from the cytoplasm in the autophagosome and (iii) the fusion of the LD-containing autophagosome (lipophagosome) with the vacuole/lysosome where (iv) the LD is finally degraded. A detailed discussion of lipophagy in general and macrolipophagy in particular is beyond the scope of the present review and can be found elsewhere [46,52]. Despite macrolipophagy being the first form of lipophagy reported in animal cells, microlipophagy is more common across the species (Figure 1). Note that both macro- and microlipophagy have also been identified in plants and algae depending upon the species under observation [53].

For some time, classical macrolipophagy was thought to be the only form of lipophagy in animal cells. However, recent studies showed that animal cells utilize the CMA-assisted macrolipophagy [54,55] and also, microlipophagy [37,56]. In the case of the CMA-dependent macrolipophagy, some perilipins (PLINs) on the LD surface are first degraded by the CMA (Figure 1, right panel). Specifically, PLIN2, 3, and 5 possess KFERQ-like sequence motifs required for the degradation by the CMA. Removing these and probably some other LD surface proteins is essential for macrolipophagy in animal cells [54,55]. It is important to mention that before LDs can be sequestered by the isolation membrane, large LDs must be converted into smaller LDs by lipolysis [57,58]. Therefore, before a classical macrolipophagy can proceed, some LD surface-associated proteins must be removed by the CMA and the non-autophagic degradation of large LDs into smaller ones needs to take place.

In summary, it can be said that in mammalian cells, LDs undergo several kinds of modifications, including changes in size and alterations in the composition of surface proteins. The process of macrolipophagy, which necessitates the removal of several LD surface proteins before it can proceed, suggests that many LD-associated proteins possess anti-lipolysis/anti-lipophagy properties or act as negative regulators of lipolysis/lipophagy. The identification of such proteins is an interesting and important area of research.

Another form of lipophagy, which is more common in fungal, plant, and algal cells (microlipophagy), is discussed in the next section.

## 3. Microlipophagy

In microlipophagy, LDs are directly taken up by vacuoles or lysosomes through the invagination or protrusion of vacuolar/lysosomal membranes (Figure 1). Unlike macrolipophagy, microlipophagy is less understood and less studied. The lesser attention given to microlipophagy is attributed to the fact that for quite some time, microlipophagy was only associated with yeast, plants, and algae, while macrolipophagy was (supposedly) a major lipophagy pathway in animal cells, thereby relegating microlipophagy as less critical in terms of clinical importance. Microlipophagy is best understood in yeast, and most of the information on microlipophagy comes from yeast species, mainly *S. cerevisiae* [6,7,59] and *K. phaffii* [36,60]. We will first discuss microlipophagy in yeast, followed by microlipophagy in plants, algae, and animals. The discussion of microlipophagy in yeast may help to better understand microlipophagy in more complex eukaryotes with the assumption that the molecular mechanism of microlipophagy is conserved across the species and that mainly the mode of induction and regulatory mechanisms may vary owing to the more complex nature of multicellular organisms.

### 3.1. Microlipophagy in Yeast

In yeast, microlipophagy remains the only form of lipophagy known to date. The first hint that yeast LDs might be degraded in the vacuole by microlipophagy came from pexophagy studies in *Yarrowia lipolytica* where along with the macropexophagy of small peroxisomes, cells executed a massive engulfment of giant LDs by a large central vacuole [61]. Most of the yeast studies on microlipophagy were performed under a limited set of conditions, such as the acute nitrogen starvation [6,36,60,62,63,64] and acute [59,64,65] or gradual [7,36,60,63,65,66,67,68,69,70,71] carbon starvation. Nitrogen or carbon starvation alone, or the depletion of both nitrogen and carbon sources together can induce microlipophagy [69]. Apart from the deficit of nutrients, in yeast, microlipophagy can also be induced by treating cells with endoplasmic reticulum (ER) stressors, such as dithiothreitol (DTT) and tunicamycin (TM) [62,72,73]. Genetic manipulations, like the deletion of the *CHO2* (phosphatidylethanolamine methyltransferase) gene, also lead to microlipophagy by creating phospholipid imbalance [62,72,73]. Vacuolar fragmentation and the fusion of vacuolar compartments (upon the treatment of cells with DTT or TM) are also a part of microlipophagy [72,73]. All these suggest that microlipophagy can be induced by different means, and microlipophagy is likely the default form of lipophagy in yeast. Surprisingly, the TOR inhibitor, rapamycin, fails to induce lipophagy in yeast, contrary to mammalian cells, where it induces macrolipophagy, suggesting that the induction of macro- and microlipophagy may involve different mechanisms, and microlipophagy may be induced independent of TOR inhibition [35,59]. It is known that cells can execute both TOR-dependent and independent autophagy. Therefore, this raises the possibility of TOR-independent microlipophagy in yeast. Whether this is true for microlipophagy in plants, algae, and animals needs further investigation.

As demonstrated above, the lack of nutrients (nitrogen and/or carbon) or the presence of ER stress (due to phospholipid imbalance, DTT, or TM) can induce microlipophagy. However, microlipophagy might be physiologically important even in rich medium by providing fatty acids to sustain cellular growth, especially if cells are unable to synthesize them de novo [6]. Yeast studies also suggested that microlipophagy might be one of the processes that prevent the Niemann–Pick type C neurological disease [63]. Microlipophagy is also required for energy production and longevity during acute glucose starvation [59]. Proper LD–vacuole contacts and microlipophagy limit the related through the vacuolar membrane protein, Vac8, micronucleophagy (i.e., microautophagy of the nucleus) [65]. Interestingly, microlipophagy is also important for the adaptation of cells to phospholipid imbalance and other ER stressors by removing unfolded ER proteins, which become associated with newly formed LDs en route to the vacuole [62,72]. Therefore, microlipophagy is an important mediator of the ER stress-induced ER proteostasis. All these functions prove that microlipophagy in yeast is physiologically relevant under growth, starvation, and stress conditions.

The vacuolar degradation of LDs during microlipophagy involves the reorganization of the vacuolar membrane into two distinct domains: sterol-rich liquid-ordered (L_o_) and sterol-poor liquid-disordered (L_d_). Apart from the lipid composition differences, these vacuolar microdomains differ significantly in protein composition and are highly dependent on the cell environment (reviewed by [51]). For example, proteins, such as Gtr2 and Ivy1, are present in the L_o_ domain, while absent in the L_d_ domain. Other proteins present in the L_o_ domain include members of the autophagy-related (Atg) group of proteins, such as Atg6 and Atg14, and other proteins, like Gtr1, Iml1, Lam6/Ltc1, Nce102, and Tco89. The formation of vacuolar microdomains involves several proteins, including Erg5, Erg6, Fab1, Lam6, Mpk1, Nce102, Nem1, Sec18, and Vps4. Other proteins required for microdomain formation include Are1/2, Ldb16, Ldo16/45, Ncr1, and Npc2 (reviewed in [51]). Just like the protein composition of vacuolar microdomains, proteins taking part in their formation differ in various cell environments. For example, Fab1, Mpk1, Nem1, and Vps4 are needed for vacuolar microdomain formation during the stationary phase, weak acid stress, and acute starvation (cells in water). In contrast, only Fab1 and Vps4 are required during the treatment of cells with cycloheximide [74]. Similarly, Lam6 is needed for vacuolar microdomain formation in cycloheximide-treated cells or cells during acute carbon starvation, but not under weak acid stress [75]. However, Gtr1, Gtr2, Iml1, Ivy1, and Tco89 are present in vacuolar microdomains, but do not contribute to their formation. Apart from the proteins mentioned above, lipid kinases, such as PtdIns 4-kinases (Pik1 and Stt4 in yeast), must also contribute to vacuolar microdomains [69,76]. Interestingly, PtdIns 4-kinases are conserved across species, suggesting their potential involvement in lipophagy in animal cells [77]. Therefore, proteins present in vacuolar microdomains, and proteins needed for vacuolar microdomain formation are regulated by the stress involved in the induction of microlipophagy. At the same time, most of the proteins present in and required for vacuolar microdomains remain the same, and only a few proteins come or go, which is dependent on how microlipophagy is induced. This makes sense because by just regulating or modulating a few proteins, cells can fine-tune microlipophagy without shaking up the entire machinery.

Once microlipophagy is induced, the complete internalization and degradation of LDs by the vacuole is another feat the cell must complete. The internalization of LDs by the vacuoles starts from the L_o_ vacuolar domain. Starvation or ER stress leads to vacuolar microdomain formation. Vacuolar microdomains, primarily the L_o_ domain, are needed for the contact between LDs and the vacuole [7,73]. Recently it was reported that Vac8 interacts with LDO (LD organization) proteins (Ldo16 and Ldo45) at the vacuole–LD contact site (vCLIP) for microlipophagy [65,71]. Once the contact between the vacuole and the LD is established, the ESCRT complex on the vacuolar membrane further helps vacuoles in the internalization of LDs [62,72,73]. Specifically, the ESCRT complex at the neck of the invagination helps in the LD capture inside the vacuole. Apart from its role in microlipophagy, the ESCRT complex is also involved in the microautophagy of the Vph1 vacuolar membrane protein [67]. Therefore, the ESCRT complex is not specific to any cargo or pathway, and many microautophagic pathways may require the ESCRT machinery. A detailed discussion on ESCRT pathways, components, regulation, and other aspects is beyond the scope of this review and can be found elsewhere [78,79,80].

So far, throughout this discussion, we have yet to talk about Atg proteins and their role in general microautophagy and microlipophagy. The role of Atg proteins in microlipophagy highly depends on how microlipophagy is induced. The involvement or the requirement of Atg proteins changes with conditions used to induce microlipophagy and may also differ with species or organisms. For example, in *K. phaffii*, core Atg proteins, including Atg1-Atg9, are essential for the nitrogen starvation-induced microlipophagy [36]. In contrast, only Atg6 is essential for the stationary phase microlipophagy, while the rest of the core Atg proteins are partially required [36]. Apart from core Atg proteins, the ubiquitin-binding autophagic receptor, Cue5, is co-degraded with LDs only during the stationary phase microlipophagy but is dispensable for its mechanism [60,81]. In the case of *S. cerevisiae*, the core Atg machinery is required during both nitrogen starvation- and stationary phase-induced microlipophagy [6,7]. In *S. cerevisiae*, core Atg proteins are also required for microlipophagy under acute glucose starvation conditions [59]. However, core Atg proteins are not required for microlipophagy during ER stress in budding yeast [62,72]. Surprisingly, Atg1 is also not required after the diauxic shift [67]. Therefore, the role of Atg proteins in microlipophagy may be indirect as it is highly dependent on species and the mode of induction of microlipophagy.

It is essential to mention that apart from autophagic proteins, there are several non-autophagic proteins that also participate in microlipophagy, depending on how lipophagy is induced. For example, Ncr1 and Npc2 are required for the nitrogen starvation-, stationary phase-, and phospholipid imbalance-induced microlipophagy, but not for the DTT- or TM-induced microlipophagy [63,73]. Also, while the ESCRT machinery is required for microlipophagy after the diauxic shift, in the stationary phase, under nitrogen starvation conditions and during ER stress, it negatively affects microlipophagy during acute glucose starvation [62,63,64,67,72].

A list of different proteins tested for their involvement in lipophagy in yeast can be found elsewhere [82]. It is also likely that we will uncover more proteins involved in yeast microlipophagy as time goes on.

### 3.2. Microlipophagy in Plants and Algae

While autophagy in plants is a well-documented phenomenon, the study of lipophagy in plants is a relatively unexplored area. Unlike extensive research on lipophagy in yeast and animals, our understanding of lipophagy in plants needs to be improved [83]. The presence of LDs in the vacuoles of the germinating seeds of *Arabidopsis thaliana* supports the concept of lipophagy in plants [84,85]. Mutations of the *ATG12* gene affecting the abundance of LDs in the cells of maize *Zea mays* further demonstrate the occurrence of lipophagy in plants [86]. In rice, LDs were found inside the vacuole in postmeiotic tapetum cells and this depended on ATG7, suggesting a lipophagy process [87]. Another study in *A. thaliana* found that LDs were internalized by vacuole in the process resembling microautophagy [45]. Just like yeast, *A. thaliana* also possesses proteins with AIM and UIM motifs needed for interaction with Atg8 [88,89]. One of such proteins, Caleosin 1 (CLO1), has been shown to be involved in microlipophagy in *A. thaliana* [90,91].

Although they are barely observed in mature leaves, LDs increase in number during both natural senescence, drought, nutrient deficit (abiotic stress), and infection by plant pathogens (biotic stress), accumulating lipids derived from the dismantlement of membranes [92,93,94]. The complicated relationship between lipophagy and diverse stress conditions known to induce lipophagy has been studied in several algal species. For example, in *Auxenochlorella protothecoides* (green alga), LDs are turned over by a mechanism resembling microlipophagy [95], while another green alga, *Micrasterias denticulata*, uses macrolipophagy [96]. Like *M. denticulate*, the green alga *Chlamydomonas reinhardtii* also uses macroautophagy to degrade LDs, but during this process, LDs fuse with autophagosomes, suggesting that this mode of degradation might also resemble crinophagy [97].

The induction of both macro- and microlipophagy in algal species suggests the possibility of both types of lipophagy in plants, similar to mammalian cells (see below). A recent study on the senescent leaves of watermelon (*Citrullus lanatus*) supports this suggestion: in the senescent leaf cells with small vacuoles, LDs are degraded by the macrolipophagy-like pathway [98]. In contrast, LDs in the senescent leaf cells with large central vacuole use the microlipophagy-like process. However, it remains unclear how this decision is made, whether the LD will be turned over by macro- or microlipophagy. Whether plant and algal cells use vacuolar membrane reorganization (to form membrane microdomains, like those in yeast) for microlipophagy also needs further research. Just like it is important in yeast, lipophagy might be crucial in plants. For example, as one of the ATG7-dependent processes, lipophagy may contribute to the postmeiotic anther development in rice [87].

### 3.3. Microlipophagy in Animals

Macrolipophagy is believed to be the predominant form of lipophagy in animal cells. However, a report published in the year 2020 by Schulze et al., for the first time, showed the induction of microlipophagy in hepatocytes [37]. Surprisingly, previous hepatocyte studies failed to detect microlipophagy and only observed macrolipophagy [35]. It was further observed that microlipophagy is independent of macrolipophagy and CMA in hepatocytes [37]. Instead, it depends on Rab5 [99]. Another recent study by Menon et al. has shown the involvement of ARL8B in mediating direct contact between LDs and lysosomes in macrophages [100]. Interestingly, microlipophagy was also reported in cardiomyocytes [38]. The authors used a high-fat diet to induce lipophagy and observed that Rab7 is essential for microlipophagy in diabetic cardiomyopathy. Finally, Liu et al. showed that the inhibition of macrolipophagy by 3-MA failed to prevent the flux of free fatty acids from LDs via lysosomes into mitochondria in beta cells, suggesting the operation of microlipophagy in yet another cell type [101].

So far, within the last 5 years, several independent studies in different cell types showed the induction of microlipophagy in animals [37,38,56,99,100,101]. Furthermore, in most cases, authors did not observe macrolipophagy, suggesting that microlipophagy was the predominant or the only form of lipophagy in examined cells or tissues. Consistently, Tong et al. failed to see LDs in autophagosomes in the cardiac cells of mice on a high-fat diet [102]. This suggests that macro- and microlipophagy can be induced separately. However, the detailed mechanism of microlipophagy in mammalian cells is missing and further research is needed. It is still unclear whether the ubiquitin-binding autophagic receptors, such as SQSTM1 [103], which are central in the macrolipophagy pathway, play any role during microlipophagy.

Despite macrolipophagic and microlipophagic bodies inside vacuoles/lysosomes having different membrane composition due to different origins of their membranes (inner autophagosomal membrane vs. vacuolar/lysosomal membrane, respectively) (Figure 1), they might share the same fate after membrane breakdown inside the lytic compartment. The neutral lipids of LDs are decomposed to fatty acids by lysosomal acid lipase and free fatty acids are exported to the extracellular environment via lysosomal exocytosis mediated by the fusion of lysosomes with the plasma membrane [104]. This process is regulated by the lysosomal calcium channel, MCOLN1, and precedes the reuptake of fatty acids by the same or neighboring cells with a potential role in their lipid exchange/signaling [104].

The studies above confirmed the idea that microlipophagy is conserved from yeast to animal cells. Therefore, animal cells can use either macro- or microlipophagy. However, what decides whether it will be macro- or microlipophagy and if the reorganization of the lysosomal membrane happens during microlipophagy in animal cells, similar to the reorganization of the vacuolar membrane in yeast, remains unknown. We summarized the organisms/species, cell types, and experimental conditions in which basal or induced microlipophagy has been observed to date in Table 1.

## 4. Microlipophagy in Cell Physiology and Diseases

As the importance and understanding of autophagy are improving daily, the role of autophagy in general and lipophagy in particular in diseases and other clinical conditions is becoming more apparent. Lipophagy has been implicated in medical conditions ranging from fatty liver [46,111] and metabolic disorders [47,52] to cancer [112,113], neurodegeneration [48], kidney disease [114], atherosclerosis [115,116], ischemic heart disease [117], and diabetes [118]. In addition to the clinical conditions mentioned above, lipophagy, which regulates the abundance of LDs in mammalian cells, also affects viral infection and its spread [119].

Apart from the importance of “lipophagy” in health and diseases mentioned above, the involvement of “microlipophagy” in diseases is becoming a topic of discussion in the community. With observing microlipophagy in animal cells, its importance in diseases cannot be ruled out. For example, the perturbation of microlipophagy can lead to critical liver diseases. This is possible because microlipophagy is the predominant mode of lipophagy in liver cells [37,120]. Consistently, another report hinted toward the role of microlipophagy in alcohol-associated hepatic steatosis [99]. Recently, microlipophagy has also been implicated in diabetic cardiomyopathy [38]. Therefore, the involvement of microlipophagy in other clinical conditions cannot be excluded. Future research will help to establish the role of microlipophagy in other diseases.

## 5. Gaps in Knowledge

After the discovery of lipophagy as a novel means of the LD degradation in cells, efforts were made to understand the detailed mechanism of lipophagy with the idea that a better understanding of the regulation of lipophagy may help treat diseases associated with LD accumulation. This catalyzed research related to lipophagy, and our knowledge of lipophagy has improved significantly over the last 15 years. However, there is still a need to better understand lipophagy in general and microlipophagy in particular (concerning the present review). The recent observation of microlipophagy in mammalian cells further challenged our understanding of lipophagy pathways. Despite significant improvements in understanding microlipophagy, some of the key gaps that need to be filled remain and are given below. Note, this list is not comprehensive, and with time, the number of questions will increase as the field of microlipophagy continues to evolve.

- How do cells decide whether to proceed with macro- or microlipophagy?
- Can macro- and microlipophagy co-occur in the same cell?
- Is the reorganization of the vacuolar membrane during microlipophagy in yeast standard in other species, including the lysosomal membrane in animals?
- Is the vacuolar membrane reorganization into microdomains also essential for other autophagic pathways, in addition to microlipophagy and micronucleophagy?
- What are the signaling pathways channeling information for the vacuolar microdomain formation during microlipophagy?
- What is precisely the role of Atg proteins during microlipophagy under different experimental conditions?
- Do the LD protein ubiquitination and ubiquitin-binding autophagic receptors contribute to the microlipophagy mechanism?
- How common is microlipophagy in animal cells? Is it cell- or tissue-specific, or can it be potentially induced in all cell types?
- Is microlipophagy a default pathway of lipophagy, or is microlipophagy happening only under specific conditions?
- Is microlipophagy more ancient and primitive compared to macrolipophagy, owing to the widespread conservation of microlipophagy?

Looking at the above points highlights the exciting challenges and opportunities in the field of lipophagy, in general, and microlipophagy in particular.

## 6. Conclusions

Based on the studies mentioned above, it can be said that, like autophagy in general, microlipophagy is also conserved across the eukaryotic kingdom. Since microlipophagy is present from unicellular yeasts to multicellular mammals, it is possible that microlipophagy originates earlier than macrolipophagy, and microlipophagy is a crude or rudimentary form of lipophagy, and macrolipophagy is a more recent event during evolution, acquired due to the increasing complexity of cells. Both macro- and microlipophagy can potentially happen in every complex eukaryotic cell, depending on environmental conditions and clues. Furthermore, the size of LDs and lytic compartments might have a role in dictating the type of lipophagic pathway due to the greater size constraints of the following: (i) lysosomes compared to autophagosomes in animal cells, and (ii) small vacuoles compared to a large central vacuole in plant cells. Undoubtedly, future studies providing additional insights from different species will help us better understand microlipophagy, its mechanisms, and physiological significance.

## Figures and Tables

**Table 1:** Organisms, cells, and conditions in which microlipophagy has been observed to date.

**Organism**	**Cell Type**	**Condition**	**Reference**
**Fungi**			
*K. phaffii*	-	Stationary phase	[36,60]
		Acute nitrogen starvation	
*M. grisea*	Appressoria	Appressorium maturation	[105]
*P. distincta*	Aeciospores	Aeciospore aging	[106]
*S. cerevisiae*	-	Diauxic shift	[67]
		Stationary phase	[7,63,65,66,68,69,70,71]
		Acute carbon starvation	[59,64,65]
		Acute nitrogen starvation	[6,62,63,64]
		Acute carbon and nitrogen starvation	[69]
		ER stress	[62,72,73]
**Plants**			
*A. thaliana*	Vegetative cells of pollen	Pollen development	[107]
	Cotyledon cells	Seed germination	[90,91]
	Cotyledon and leaf cells	Dark-induced starvation	[45]
*C. lanatus*	Central vacuole-containing cells	Leaf senescence	[98]
**Algae**			
*A. protothecoides*	-	Heterotrophy–autotrophy transition	[95]
*C. reinhardtii*		Nitrogen resupply	[108]
*N. oceanica*			[109]
*P. kessleri*		Salt stress	[110]
**Animals**			
*H. sapiens*	THP1 monocytes	Post differentiation into macrophages	[100]
	Primary PBMCs		
	HepG2 and Hep3B cells	Regular medium	[99]
*M. musculus*	AML12 hepatocytes	Serum starvation	[37]
	Cardiomyocytes	High-fat diet	[38]
*P. sinensis*	Leydig cells	Hibernation period	[56]
*R. norvegicus*	Primary hepatocytes	Serum starvation	[37]
		From fed rat livers	[99]
	INS1 beta cells	PLIN2 knockdown	[101]

## Data Availability

No new data were created or analyzed in this study.
